# Vascular disease and vascular risk factors in relation to motor features and cognition in early Parkinson's disease

**DOI:** 10.1002/mds.26698

**Published:** 2016-10-06

**Authors:** Naveed Malek, Michael A. Lawton, Diane M. A. Swallow, Katherine A. Grosset, Sarah L. Marrinan, Nin Bajaj, Roger A. Barker, David J. Burn, John Hardy, Huw R. Morris, Nigel M. Williams, Nicholas Wood, Yoav Ben‐Shlomo, Donald G. Grosset, on behalf of the PRoBaND Clinical Consortium

**Affiliations:** ^1^ Department of Neurology, Institute of Neurological SciencesQueen Elizabeth University HospitalGlasgowUnited Kingdom; ^2^ School of Social and Community MedicineUniversity of BristolBristolUnited Kingdom; ^3^ Institute of NeuroscienceUniversity of NewcastleNewcastle upon TyneUnited Kingdom; ^4^ Department of NeurologyQueen's Medical CentreNottinghamUnited Kingdom; ^5^ Department of Clinical NeurosciencesJohn van Geest Centre for Brain RepairCambridgeUnited Kingdom; ^6^ Reta Lila Weston Laboratories, Department of Molecular NeuroscienceUCL Institute of NeurologyLondonUnited Kingdom; ^7^ Department of Clinical NeuroscienceUCL Institute of NeurologyLondonUnited Kingdom; ^8^ Institute of Psychological Medicine and Clinical Neurosciences, MRC Centre for Neuropsychiatric Genetics and GenomicsCardiff UniversityCardiffUnited Kingdom; ^9^ Department of Molecular NeuroscienceUCL Institute of NeurologyLondonUnited Kingdom

**Keywords:** Parkinson's disease, gender, phenotype, diabetes, cerebrovascular

## Abstract

**Objective:**

The purpose of this study was to examine the relationship between vascular disease (and vascular risk factors), cognition and motor phenotype in Parkinson's disease (PD).

**Methods:**

Recently diagnosed PD cases were enrolled in a multicenter prospective observational longitudinal cohort study. Montreal cognitive assessment (normal >23, mild cognitive impairment 22 to 23 or lower but without functional impairment, and dementia 21 or less with functional impairment) and Movement Disorder Society Unified PD Rating Scale part 3 (UPDRS 3) scores were analyzed in relation to a history of vascular events and risk factors.

**Results:**

In 1759 PD cases, mean age 67.5 (standard deviation 9.3) years, mean disease duration 1.3 (standard deviation 0.9) years, 65.2% were men, 4.7% had a history of prior stroke or transient ischemic attack, and 12.5% had cardiac disease (angina, myocardial infarction, heart failure). In cases without a history of vascular disease, hypertension was recorded in 30.4%, high cholesterol 27.3%, obesity 20.7%, diabetes 7.2%, and cigarette smoking in 4.6%. Patients with prior stroke or transient ischemic attack were more likely to have cognitive impairment (42% vs 25%) and postural instability gait difficulty (53.5% vs 39.5%), but these findings were not significant after adjustment for age, sex, and disease duration (*P* = .075). The presence of more than 2 vascular risks was associated with worse UPDRS 3 motor scores (beta coefficient 4.05, 95% confidence interval 1.48, 6.61, *p* = .002) and with cognitive impairment (ordinal odds ratio 2.24, 95% confidence interval 1.34, 3.74, *p* = .002). In 842 patients (47.8%) with structural brain imaging, white matter leukoaraiosis, but not lacunar or territorial infarction, was associated with impaired cognition (*p* = .006) and postural instability gait difficulty (*p* = .010).

**Conclusion:**

Vascular comorbidity is significantly associated with cognitive and gait impairment in patients with early PD, which may have prognostic and treatment implications. © 2016 The Authors. Movement Disorders published by Wiley Periodicals, Inc. on behalf of International Parkinson and Movement Disorder Society.

Cognitive impairment and dementia are recognized consequences of the evolving neurodegenerative processes underlying Parkinson's disease (PD) and represent a significant management issue. Impaired cognition is increasingly recognized in early PD, graded as mild cognitive impairment in 14.2% and dementia in 16.3% of cases in one study (N = 492) within 3.5 years of diagnosis.^1^ In another study that excluded cases with dementia, 34% had mild cognitive impairment (MCI) at an average patient age of 61 years and a mean disease duration of 3.4 years at the time of their first assessment.^2^ However, cognitive impairment and dementia in PD may also relate to comorbid cerebrovascular disease. Clinical, imaging, and pathological studies highlight the adverse impact of cerebrovascular disease and vascular risk factors including diabetes, hypertension, and dyslipidemia on cognition and motor tasks (particularly gait).^3^, ^4^, ^5^, ^6^ Because the prevalence of cerebrovascular disease and vascular risk factors increases with age, particularly in high‐income countries, it seems likely that they contribute to cognitive impairment and motor disability in PD.

Gait impairment and falls result, in part, from motor dysfunction in PD and are more likely in patients with axial involvement, recognized clinically as the postural instability gait difficulty (PIGD) motor phenotype, distinct from the tremor dominant (TD) motor phenotype.^7^ An association of the PIGD phenotype with cognitive impairment is well documented in PD,^8^ and gait impairment is common after ischemic stroke but also occurs in the absence of acute cerebrovascular events.^9^, ^10^ Specifically, in PD, subclinical cerebrovascular disease was linked to greater motor severity and increased gait impairment in two small, but detailed, studies that included structural MRI and functional dopaminergic imaging.^11^, ^12^ Axial impairment increased in relation to the white matter cerebrovascular burden with a stronger relationship than that between white matter changes and bradykinesia, and there was no relationship with either tremor or rigidity.^12^ An overlap syndrome between PD and cerebrovascular disease may therefore create a mixed motor phenotype and explain the limited responsiveness of some of these motor and cognitive features to antiparkinsonian therapy.

Because some of these risk factors are modifiable,^13^ reducing the direct effects of ischemia‐related neuronal loss would be one aim of this approach to limit the damaging effects from comorbid cerebrovascular disease on cognition and gait. Progression of such cognitive and gait problems in PD occurs on average at 6.2 years from diagnosis to dementia and 7.1 years from diagnosis to falls in prospective studies.^14^, ^15^ A window of opportunity may therefore exist around the time of diagnosis of PD, or earlier, considering current research efforts in identifying premotor or preclinical PD. Although there are wider population‐based initiatives looking at preventive approaches for vascular disease to reduce vascular dementia rates, the issues in PD may be even more pertinent. More specific interaction between vascular risk factors and PD have been proposed that involve acceleration of the neurodegenerative process, particularly in the presence of diabetes.^16^

Our objective was to test the hypothesis that vascular and metabolic factors are associated with cognitive and motor features in recent onset PD, with the aim of explaining differences in PD phenotype that arise from these comorbidities.

## Methods

*Tracking Parkinson’s* study is a large, prospective, observational, multicenter project in the United Kingdom. Patients were recruited with a clinical diagnosis of PD, fulfilling Queen Square Brain Bank criteria^17^ and supported by structural and/or functional neuroimaging performed when the diagnosis was not firmly established clinically. Both drug‐naïve and ‐treated patients aged 18 to 90 years were eligible. All cases were diagnosed with PD in the preceding 3.5 years, and recruitment was completed between February 2012 and May 2014. Patients were excluded in the presence of severe comorbid illness, other degenerative forms of parkinsonism (eg, progressive supranuclear palsy), or symmetrical lower body parkinsonism attributable to significant cerebrovascular disease (patients with incidental vascular disease on brain imaging were not excluded). Patients with drug‐induced parkinsonism were excluded, but drug‐unmasked PD was allowed if justified by abnormal functional dopaminergic imaging. Patients with a clinical diagnosis of dementia at their first assessment were also excluded. Patients were enrolled in a 6‐month follow‐up, but only results from the baseline visit are reported in this article. Enrolled patients whose diagnosis was later changed, on clinical or imaging grounds, were excluded from the analysis. In addition, patients with missing data or in whom there were atypical features that might indicate an alternative diagnosis, including those with a minimal response to dopaminergic therapy, were excluded from the main analysis.

The study was carried out in accordance with the Declaration of Helsinki. Research funding for this project is from Parkinson's UK, the national patient care and research organization.

A total of 72 sites in the United Kingdom providing secondary care treatment for PD patients as part of the UK National Health Service (and in selected sites, their linked academic institutions) participated, with multicenter ethics committee and local research and development department approvals. All participating patients provided written informed consent at the time of recruitment.

Clinical assessments were made at baseline using standardized and validated scales to document the motor and nonmotor features and quality of life of the enrolled patients. Levodopa equivalent daily dose was calculated using established formulae for dose equivalence.^18^ Motor subtypes were determined using the Movement Disorder Society Unified Parkinson's Disease Rating Scale part 3 (UPDRS 3) scores using a predetermined formula.^7^ Motor scoring was performed without stopping antiparkinsonian medication, and the motor state was recorded as either being “on” or “off” (although such fluctuations are rare at this stage of disease). Montreal cognitive assessment (MoCA) scores were adjusted for years of education. Predetermined diagnostic cut‐offs were used to categorize cases into normal (>23) mild cognitive impairment (MCI) 22‐23 or less than 22 but without functional impairment and dementia (21 or less with functional impairment) to reflect core criteria for PD dementia defined by the Movement Disorder Society Task Force.^19^

Prior medical histories were recorded by the patients, often with corroboration from a spouse or caregiver, including previous histories of stroke, transient ischemic attack (TIA), or cardiac disease (angina, myocardial infarction, or heart failure). Neuroimaging was performed on clinical grounds (1.5 or 3T), and findings were categorized by visual reporting as revealing lacunar or territorial infarction and/or periventricular/subcortical white matter hyperintensities (leukoaraiosis). For overall assessment of vascular disease risk, we calculated QRISK2, which encompasses risk factors including age, gender, elevated cholesterol, blood pressure/treatment, diabetes, smoking status, body mass index, and chronic kidney disease and is appropriate in patients who have not had a prior vascular event.^20^

Furthermore, we searched PubMed and the Cochrane Database up to November 1, 2015, combining the search terms “vascular,” “leukoaraiosis,” “diabetes,” “hypertension,” “stroke,” and “Parkinson's” to look for any other similar studies so that our research could be put into context and our results compared with other studies.

### Statistical Analysis

The main analysis was performed without imputation of missing data in the 1759 patients with available data and without atypical features. Additional analyses were undertaken in 2 ways.

First, imputation was performed with the main data set (*N* = 1759). We used imputation methods to adjust for missing outcomes and exposure data. For MoCA, motor phenotype, and UPDRS 3, we first calculated expected scores where at least 80% of the responses were available by up‐weighting the score based on answered questions (eg, when 30 of 33 questions were answered, the score was uprated by multiplying by 33/30). Any remaining missing data were imputed using the chained equation approach to multiple imputation, creating 10 imputed data sets. MoCA scores and UPDRS scores were imputed using predictive mean matching and motor phenotype using multinomial logistic regression. Estimates and *P* values were derived from the 10 datasets using Rubin's rules.^21^

Second, analyses were performed without imputation (n = 1930). We applied the methodology used for data analysis in the main dataset (n = 1759) to the bigger dataset (ie, including cases with one or more atypical features).

Phenotypic characteristics requiring covariate adjustment were analyzed using multivariable regression. For UPDRS 3 scores, standard linear regression was used for categorized MoCA ordered logistic regression (also called a proportional odds model) and for motor phenotype multinomial logistic regression with tremor dominant as the baseline. We adjusted for levodopa equivalent daily dose in our models using UPDRS 3 as the dependent variable and for drug naiveté in the analyses for cognitive impairment. For analysis of the association between categorized neuroimaging results and the different outcomes, heterogeneity *P* values across the three groups were calculated (ie, a hypothesis test that all three groups are equivalent with regards to the outcome). All *P* values were 2‐tailed; *P* values were calculated before and after adjustment for potential confounders. Statistical analysis was conducted using STATA (version 13, StataCorp, College Station, Texas).

## Results

**Figure 1:**
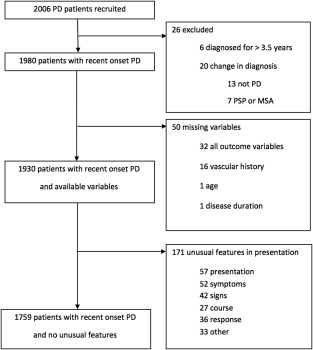
CONSORT flow diagram showing the disposition of cases recruited to the study and reasons for exclusion from the main analysis dataset of 1759 patients. Additional analyses were undertaken using multiple imputations for missing data in the 1759 cases and on the full dataset of 1930 cases with available data (see text).

There were 2006 patients recruited, of whom 247 (12.3%) were excluded for the following reasons: change in diagnosis (during a mean follow‐up from baseline of 2.6 years, SD 0.6); protocol violation; missing data (which affected 6.7% to 8.0% for outcomes, but taking into account those who answered at least 80% of the questions it ranged from 0.3% to 2.8%); or possible atypical features raising diagnostic doubt (Fig. 1). The main analysis group therefore consisted of 1759 PD cases, mean age was 67.5 (SD 9.3) years, mean disease duration 1.3 (SD 0.9) years, and 65.2% were male (Table 1). Of the patients, 4.7% had a prior history of stroke or TIA, and 12.5% had cardiac disease (angina, myocardial infarction, heart failure; Table 2). Vascular risk factors (in those without a history of stroke, TIA, or cardiac disease) were hypertension 30.4%, high cholesterol 27.3%, obesity 20.7%, diabetes 7.2%, and cigarette smoking 4.6%. With regard to the exposures, missing data were less than 2.5% for all variables except smoking that had 12.4% missing data.

**Table 1:** Demographic and motor profile in 1759 cases of recent onset PD and no unusual presentation features

Characteristic		Total N (%) or mean (SD)
Age in years	Onset/Diagnosis/At baseline	64.3 (9.8)/66.1(9.3)/67.5 (9.3)
Gender (male)		1147 (65.2)
Disease duration in years		1.3 (0.9)
Race, White		1720 (98.2)
Symptoms at onset	Tremor/Rigidity/Bradykinesia/Postural instability	1304 (75.6)/1181 (71.9)/1309 (78.0)/306 (18.8)
Motor subtype	TD/PIGD/Indeterminate	765 (47.0)/653 (40.1)/209 (12.9)
UPDRS 3		22.5 (12.1)
Hoehn and Yahr stage	0/1/2/3/4/5	5 (0.3)/863 (49.7)/767 (44.2)/97 (5.6)/4 (0.2)/1 (0.1)
Drug naïve		172 (9.8)
LEDD (mg/day)		293 (205)

PD, Parkinson's disease; SD, standard deviation; TD, tremor dominant; PIGD, postural instability gait difficulty; UPDRS 3, Movement Disorder Society Unified Parkinson's Disease Rating Scale Part 3; LEDD, levodopa equivalent daily dose.

**Table 2:** Motor and cognitive profile classified by prior history of stroke or cardiac disease, in cases with no unusual presentation features

	Previous stroke or TIA, N (%)		Model estimates^d^ (95% CI)	p value^d^	Cardiac disease, N (%)		Model estimates^d^ (95% CI)	*P* value^d^
Characteristic	Yes	No			Yes	No		
	83 (4.7)	1674 (95.3)			218 (12.5)	1532 (87.5)		
Montreal cognitive assessment^a^								
Normal	47 (58.0)	1170 (75.0)	1.53^e^ (0.96, 2.45)	0.075^f^	132 (63.2)	1080 (75.7)	1.22^e^ (0.89, 1.68)	.22^f^
MCI	31 (38.3)	349 (22.4)			67 (32.1)	313 (21.9)		
Dementia	3 (3.7)	40 (2.6)			10 (4.8)	33 (2.3)		
UPDRS 3^b^	25.5 (12.0)	22.3 (12.1)	1.37^g^ (−1.45, 4.20)	0.34^h^	25.4 (12.8)	22.1 (11.9)	1.78^g^ (−0.04, 3.60)	.055^h^
Motor phenotype^c^								
TD	25 (35.2)	739 (47.5)	1^i^ (ref)		75 (38.3)	687 (48.2)	1^i^ (ref)	
PIGD	38 (53.5)	615 (39.5)	1.61^i^ (0.95, 2.72)	0.075	94 (48.0)	555 (39.0)	1.37^i^ (0.98, 1.92)	.065
Indeterminate	8 (11.3)	201 (12.9)	1.19^i^ (0.52, 2.69)	0.68	27 (13.8)	182 (12.8)	1.41^i^ (0.87, 2.29)	.17

TIA, transient ischemic attack; CI, confidence interval; MCI, mild cognitive impairment; UPDRS 3, Movement Disorder Society Unified Parkinson's Disease Rating Scale Part 3; TD, tremor dominant; PIGD, postural instability gait difficulty.

a Ordinal logistic regression model (normal = 0, MCI = 1, dementia = 2).

b Linear regression model.

c Multinomial logistic regression model with TD as baseline.

d Adjusted for age, gender, and disease duration.

e Odds ratio.

f Also adjusted for drug naïve.

g Beta coefficient (adjusted difference in means).

h Also adjusted for levodopa equivalent daily dose.

i Multinomial odds ratio.

Diabetes was significantly associated with increased motor severity (*P* = .006). Those with diabetes had a UPDRS 3 score that was approximately 3.7 points higher (95% confidence interval [CI] 1.07, 6.22) than those without diabetes (Table 3). There was no association between diabetes and PIGD (data not shown). The presence of multiple (>2) vascular risk factors was also significantly associated with UPDRS 3 scores (*P* = .002; Table 3). QRISK2 score >20 was associated with higher UPDRS 3 scores (*P* < .001; Table 3), further confirming this association between vascular risk factors and motor severity. Our sensitivity analysis excluding drug naïve patients found very similar associations (results not shown).

**Table 3:** Motor severity in recent onset PD, in relation to vascular risk factors, restricted to 1483 cases without a history of stroke, TIA, or cardiac disease

	UPDRS 3^a^			
	Number (%)	Mean (SD)	Beta^b^ (95% CI)	*P* value
Vascular risk factors				
Cigarette smoking	60 (4.6)	22.5 (14.8)	2.20 (−0.88, 5.28)	.16^c^
Hypertension	449 (30.4)	23.7 (11.9)	0.97 (−0.57, 2.51)	.22^c^
High cholesterol	404 (27.3)	23.0 (12.1)	−0.38 (−1.96, 1.20)	.64^c^
Diabetes mellitus	106 (7.2)	27.3 (14.8)	3.65 (1.07, 6.22)	.006^c^
BMI > 30	300 (20.7)	23.4 (11.7)	1.58 (−0.06, 3.22)	.060^c^
Number of vascular risk factors^d^				
None	568 (44.2)	20.2 (10.7)	0 (ref)	.12
1	409 (31.9)	21.8 (12.5)	1.20 (−0.32, 2.72)	.29
2	213 (16.6)	22.0 (11.7)	1.01 (−0.87, 2.90)	.002
>2	94 (7.3)	25.2 (11.1)	4.05 (1.48, 6.61)	
Vascular risk score, QRISK2 > 20	552 (37.6)	24.6 (12.6)	3.41 (1.62, 5.20)	<.001

PD, Parkinson's disease; TIA, transient ischemic attack; UPDRS 3, Movement Disorder Society Unified PD Rating Scale Part 3; SD, standard deviation; CI, confidence interval; BMI, body mass index.

a Linear regression model, UPDRS score for all patients was mean 21.9 (SD 11.9)

b Adjusted for age, gender, disease duration, and levodopa equivalent daily dose; result is from comparison of cases with vascular risk to those without.

c Also mutually adjusted for all vascular risk factors.

d Restricted to complete cases and formal test of linear trend versus heterogeneity gave *P* value of .36 (unadjusted analysis).

Cases with more than 2 vascular risk factors were significantly more likely to have cognitive impairment (*P* = .002; Table 4).

**Table 4:** Cognitive status in recent onset PD, in relation to vascular risk factors, restricted to 1483 cases without a history of stroke, TIA, or cardiac disease.

	Cognitive status^a^				
	Normal, N (%)	MCI, N (%)	Dementia, N (%)	OR^b^ (95% CI)	
Total	1052 (76.4)	294 (21.4)	31 (2.3)		*P* value
Vascular risk factors					
Cigarette smoking	45 (78.9)	12 (21.1)	0 (0.0)	1.58 (0.79, 3.13)	.19^c^
Hypertension	302 (71.7)	108 (25.7)	11 (2.6)	1.01 (0.72, 1.40)	.97^c^
High cholesterol	258 (69.7)	103 (27.8)	9 (2.4)	1.30 (0.94, 1.81)	.12^c^
Diabetes mellitus	58 (61.7)	27 (28.7)	9 (9.6)	1.52 (0.89, 2.58)	.12^c^
BMI > 30	209 (74.9)	60 (21.5)	10 (3.6)	1.26 (0.88, 1.81)	.22^c^
Number of vascular risk factors^d^					
None	437 (82.5)	88 (16.6)	5 (0.9)	1 (ref)	.31
1	296 (78.3)	74 (19.6)	8 (2.1)	1.19 (0.85, 1.68)	.46
2	155 (76.7)	44 (21.8)	3 (1.5)	1.16 (0.77, 1.75)	.002
>2	54 (64.3)	28 (33.3)	2 (2.4)	2.24 (1.34, 3.74)	
Vascular risk score, QRISK2 >20	334 (65.1)	159 (31.0)	20 (3.9)	1.43 (0.98, 2.08)	.064

PD, Parkinson's disease; TIA, transient ischemic attack; MCI, mild cognitive impairment; OR, odds ratio; CI, confidence interval; BMI, body mass index.

a Ordinal logistic regression model.

b Adjusted for age, gender, disease duration, and drug naïve.

c Also mutually adjusted for all vascular risk factors.

d Restricted to complete cases.

Structural brain imaging was performed in 842 cases (47.9% of 1759 cases; Table 5). Cognitive impairment was more common in patients with white matter leukoaraiosis, in whom any degree of cognitive impairment was recorded in 44.6%, versus 23.7% in those with lacunar or territorial infarcts and 22.9% in those with no vascular disease on imaging (*P* = .006). The odds ratio for cognitive impairment for those with leukoaraiosis only compared with those with no vascular disease on imaging was 1.88, 95% CI 1.21, 2.91.

**Table 5:** Cognitive and motor severity in 842 cases with structural brain imaging

Characteristic	Leukoaraiosis only, N (%)	Brain CT or MR result Lacunar or territory infarct, N (%)	No vascular disease, N (%)	Model estimate^d^ Leukoaraiosis only versus no vascular disease (95% CI)	Model estimate^d^ Lacunar or territory infarct vs no vascular disease (95% CI)	*P* value^d^, ^e^
Total	121 (14.4)	100 (11.9)	621 (73.8)			
Montreal cognitive assessment^a^						
Normal	61 (55.5)	71 (76.3)	447 (77.1)	1.88^f^ (1.21, 2.91)	0.78^f^ (0.46, 1.33)	.006^g^
MCI	39 (35.5)	21 (22.6)	116 (20.0)			
Dementia	10 (9.1)	1 (1.1)	17 (2.9)			
UPDRS 3^b^	23.9 (11.3)	23.7 (12.4)	21.4 (11.4)	1.31^h^ (‐1.09,3.71)	1.74^h^ (‐0.88, 4.36)	.30^i^
Motor phenotype^c^						
TD	33 (28.9)	43 (49.4)	249 (43.2)	1^j^ (ref)	1^j^ (ref)	
PIGD	70 (61.4)	34 (39.1)	247 (42.8)	1.81^j^ (1.14, 2.88)	0.72^j^ (0.44, 1.18)	.010
Indeterminate	11 (9.6)	10 (11.5)	81 (14.0)	0.88^j^ (0.42, 1.86)	0.65^j^ (0.31, 1.37)	.53

Results based on analysis where individuals with unusual presentation were not included.

CT, computed tomography; MR, magnetic resonance; CI, confidence interval; MCI, mild cognitive impairment; UPDRS 3, Movement Disorder Society Unified PD Rating Scale; TD, tremor dominant; PIGD, postural instability gait difficulty.

a Ordinal logistic regression model (normal = 0, MCI = 1, dementia = 2).

b Linear regression model.

c Multinomial logistic regression model with TD as baseline.

d Adjusted for age, gender, and disease duration.

e Heterogeneity test *P* value across the three groups.

f Odds ratio.

g Also adjusted for drug naïve.

h Beta coefficient (adjusted difference in means).

i Also adjusted for levodopa equivalent daily dose.

j Multinomial odds ratio.

Furthermore, more cases with the PIGD motor phenotype (61.4%) were seen in those with leukoaraiosis than those with either lacunar or territorial stroke (39.1%) or no vascular disease on imaging (42.8%; *P* = .01). The odds ratio for PIGD for those with leukoaraiosis only compared with those with no vascular disease on imaging was 1.81, 95% CI 1.14, 2.88 (Table 5).

Analysis of the relationships between vascular disease and risk factors, and motor and cognitive severity and pattern, in the 1759 patients after multiple imputation showed some differences from the nonimputed datasets (Supporting Information Tables 1 to 4). The association between PIGD motor phenotype and history of stroke was significant (*P* = .018) and also between UPDRS 3 and history of cardiac disease (*P* = .034). The association between number of vascular risk factors and UPDRS 3 was now significant for 1 (*P* = .049) and 2 (*P* = .030) vascular risk factors, and remained significant for >2 risk factors (*P* < .001). All other significant associations seen in the nonimputed dataset remained significant after data imputation. In an analysis of the 1930 cases (ie, including those with one or more atypical features), associations were almost identical to those in the main analysis, but there was an additional significant association between diabetes and cognitive impairment (odds ratio 1.90, CI 1.16, 3.11, *P* = .011), which was not found in either the main or the imputed analyses. Similar to the imputed analysis, we also found a significant association between obesity and UPDRS 3 (*P* = .031).

## Discussion

The association of vascular risk factors and the phenotypic expression of PD has until now been the subject of few studies and then only involving small patient numbers.^11^, ^12^ Cerebrovascular disease (macroscopic infarcts, micro‐infarcts, and arteriolosclerosis) is common in the pathogenesis of mild parkinsonian signs, especially parkinsonian gait, particularly in the elderly.^22^ Furthermore, there is a significant association between impaired cognition and cerebrovascular disease.^23^ Finally, both cerebrovascular disease and PD are predictors for the development of the motoric cognitive risk syndrome, a newly described predementia syndrome characterized by slow gait and cognitive complaints.^24^, ^25^ In this large prospective longitudinal study of patients with recent onset PD, we found that cognitive impairment was more prevalent in those with multiple vascular risk factors. There are observations of cognitive impairment and dementia in diabetic patients in the general population, with emerging evidence that interactions between several vascular risk factors are linked to target organ damage.^3^, ^11^, ^26^, ^27^, ^28^

Cerebrovascular disease can have a role in modifying the phenotype and progression of PD. Vascular pathology in PD includes fragmentation of capillaries and damage to the capillary network in multiple brain regions, but particularly in the substantia nigra, middle frontal cortex, and brain stem nuclei. Thus, treatments that prevent vascular degeneration may improve vascular remodelling in the brain and provide a novel target to ameliorate the disease burden in PD.^29^

The presence of leukoaraiosis on structural brain imaging in the present study was associated with significantly greater baseline prevalence of cognitive impairment than was seen when imaging showed lacunar or territorial infarction or normal brain imaging results. Perhaps an explanation for this could be that not all acute infarcts affect areas of the brain subserving major cognitive functions. On the other hand, the burden of leukoaraiosis (or the equivalent descriptions of white matter change/MRI T2 hyperintensity) as seen on brain imaging relates generally to impaired cognitive performance in the nondisabled elderly as well as more specifically in PD. These 2 findings are consistent with each other but neither explains the other. Although leukoaraiosis is often a marker of small vessel cerebrovascular disease, other pathological processes such as inflammation may cause a similar appearance and could offer an alternative explanation to our findings.^3^, ^12^, ^30^, ^31^ An earlier detailed review of literature in this area summarized the findings of 11 studies; 8 of these described a significant association between leukoaraiosis and impaired cognition in PD, but 3 found no such association.^32^ Furthermore, although vascular disease and vascular risk factors may be linked to impaired cognition in PD, all persons with vascular risk factors are not cognitively impaired. In our study, 64.3% of the patients with >2 vascular risk factors had normal cognitive status. In keeping with those prior studies,^33^, ^34^, ^35^, ^36^, ^37^, ^38^ we used predefined cut‐offs for the definition of cognitive state but included a requirement for impairment of function to define dementia as required by core criteria of the Movement Disorder Society Task Force.^19^

Our search of the literature did not find any large‐scale studies (n > 400) that specifically evaluated the association of vascular disease (and vascular risk factors) with motor features and cognition in early PD. Previous studies have shown that vascular risk factors and cerebrovascular disease are common in PD patients, possibly because of their older age. A study involving 148 patients with PD at about 6 years disease duration (of whom 15 had diabetes) found that diabetes mellitus was independently associated with more severe cognitive impairment in PD, likely through mechanisms other than disease‐specific neurodegeneration.^39^ Another study involving 62 patients suggested that the severity of leukoaraiosis on MRI imaging is significantly associated with UPDRS total scores and motor scores.^40^ This argument was substantiated in another critical review that concluded that white matter leukoaraiosis was associated with worsening axial motor performance, independent of the degree of nigrostriatal dopaminergic denervation.^41^ These small studies suggest that comorbid cerebrovascular disease and associated vascular risk factors can be linked to the phenotype of PD but have not defined the prevalence or severity of such problems in early PD.

Motor severity was greater in our PD patients in the presence of diabetes, which has also been reported in a case‐control study in recent onset PD.^42^ Leukoaraiosis on imaging was associated with the PIGD motor phenotype, which is consistent both with the previous observations relating such imaging changes to posture and gait problems in the general population and with prior clinical‐imaging studies in PD.^9^, ^10^, ^12^, ^31^ However, we did not find an association between the presence of diabetes and the PIGD phenotype unlike a prior report.^11^ Our study is much larger than this other report, which included only 13 patients with PD and diabetes, and our cases were also seen much earlier in their disease course (1.3 years into their illness vs 6.9 years on average). The known evolution of the tremor dominant motor phenotype toward PIGD in PD^8^ may explain this and will be tested in our cohort with further follow‐up.

There are certain limitations to our study design. The hospital‐based setting of our study may have resulted in selection bias for structural brain imaging based on clinical decisions, which is likely to overestimate the general prevalence of cerebrovascular disease in patients with PD. On the other hand, patients with major comorbidities were ineligible for study entry, which may result in the underrepresentation of significant comorbid vascular disease. Multiple statistical tests were performed, and no adjustment was made to the significance levels to account for this. We had some missing data that might affect the validity of the results given that we applied an 80% threshold. However, sensitivity analysis of imputed data confirmed the findings in the primary analysis, and if we apply a 90% threshold, rates of missing data were also low: MoCA 1.4%, UPDRS 3 1.0%, and motor phenotype 4.5%.

The diagnosis of PD in our cohort was primarily clinical, which is subject to known error rates even when applying specific diagnostic criteria, especially in early disease.^17^ However, structural imaging was applied in 842 cases (47.9%) and functional dopaminergic imaging largely in cases of clinical diagnostic uncertainty (440 cases, 25.0%). In addition, cases receiving an alternative diagnosis during follow‐up and cases with one or more features that might indicate an alternative diagnosis were also excluded from the main analysis. The structural brain imaging protocols varied according to site, and analysis was by visual assessment. Quantitative methods and additional imaging modalities, such as ultrasound,^43^ may have provided more detail but were beyond the scope of this study. Although we cannot exclude the possibility that some of our cases may evolve to an alternative diagnosis, our cohort is representative of what is clinically diagnosed and managed as early PD. The response to dopaminergic treatment was recorded prospectively in the study, and little or no response to such treatment was one of the factors used to exclude cases from the current analysis to define a levodopa‐responsive PD patient cohort. Additional analysis performed in the larger patient group, including those with atypical features, replicated the significant associations seen in the main, more diagnostically definite analysis group. Our findings are therefore relevant to clinical practice and to research studies including early treatment trials that generally enroll similar population cohorts from clinical settings.

We obtained UPDRS motor scores during routine clinic visits without stopping antiparkinsonian medication, which may result in a mixture of “on” and “off” state scorings, although at this early stage of disease these on‐off effects are seldom significant or even evident. Of the 1624 (of 1759) for whom this was recorded, only 130 were in the “off” state, of which 44 were not on medication. Hence, this issue affected only a small proportion (5.3%) of individuals. On‐off state therefore could not have biased our results to any major degree.

We do plan to collect motor scores during a practically defined “off” state at a disease duration of 3.5 years or longer. This will add quantitative information on treatment responsiveness in future reports from this study.

Disease heterogeneity is well recognized in PD and is increasingly considered as a factor in the design, conduct, and interpretation of interventional research studies.^44^ We found significant age differences and an association of vascular comorbidity with the phenotypic expression of PD, which exemplify these problems and have implications for therapeutic trials. These observations collectively suggest that clinical trials should include an assessment of vascular risk to balance treatment groups taking account of the presence of vascular factors and to stratify subgroups because outcome measures associated with vascular risk may not be improved by any Parkinson's disease modifying effect.^44^ The mechanistic linkage of vascular problems with 2 adverse outcomes, cognitive and gait impairments, which are resistant to standard antiparkinsonian treatments, deserve further study.

Future trials to manage vascular and metabolic risk factors more aggressively deserve consideration because they may have an additional benefit on PD disease progression over and above any cardiovascular benefits.

## Authors' Roles

1. Research project: A. Conception, B. Organization, C. Execution; 2. Statistical Analysis: A. Design, B. Execution, C. Review and Critique; 3. Manuscript: A. Writing of the first draft, B. Review and Critique.

N.M.: 1B, 1C, 2A, 2C, 3A, 3B

D.M.A.S.: 1C, 3B

S.L.M.: 1C, 3B

M.A.L.: 1C

N.B.: 1C, 3B

R.A.B.: 1C, 3B

J.H.: 1B

N.W.: 1B

N.M.W.: 1B

D.J.B.: 1B, 1C, 3B

H.R.M.: 1B, 1C, 3B

Y.B.S.: 1B, 2A, 3B

K.A.G.: 1B, 1C, 2C, 3B

D.G.G.: 1B, 1C, 2C, 3A, 3B

## Full financial disclosures of all authors for the past 12 months

S.L.M. has received an honorarium from Britannia and research grant support from Parkinson's UK, Glaxo Smith Kline (GSK), and the Michael J. Fox Foundation. N.B. has received payment for advisory board attendance from UCB, Teva Lundbeck, Britannia, GSK, Boehringer, and honoraria from UCB Pharma, GE Healthcare, Lily Pharma, Medtronic. He has received research grant support from GE Healthcare, Wellcome Trust, Medical Research Council, Parkinson's UK, and the National Institute for Health Research. R.A.B. has received grants from Parkinson's UK, NIHR, Cure Parkinson's Trust, Evelyn Trust, Rosetrees Trust, Medical Research Council (MRC), and EU along with payment for advisory board attendance from Oxford Biomedica and Living Cell Technologies (LCT) and honoraria from Wiley and Springer. D.J.B. has received grants from NIHR, Wellcome Trust, GlaxoSmithKline Ltd, Parkinson's UK, and the Michael J. Fox Foundation. He has acted as consultant for GSK. J.H. has received honoraria from Eisai and grant support from MRC/Wellcome, Parkinson's UK, and the Michael J. Fox Foundation. H.R.M. has received grants from Medical Research Council UK, Wellcome Trust, Parkinson's UK, Ipsen Fund, Motor Neurone Disease Association, Welsh Assembly Government, Progressive Supranuclear Palsy (PSP) Association, CBD Solutions, and the Drake Foundation and payment for advisory board attendance and lectures from Teva, AbbVie, Boehringer Ingelheim, and GSK. D.G.G. has received payment for advisory board attendance from AbbVie and honoraria from UCB Pharma, GE Healthcare, and Civitas Inc. N.M., D.M.A.S., K.A.G., M.A.L., N.L.W., and Y.B‐S. report no conflicts of interest.

## Supporting information

Additional Supporting Information may be found in the online version of this article at the publisher's web‐site.

Table 2. Motor and cognitive profile classified by prior history of stroke or cardiac disease. Results based on sensitivity analysis where individuals with unusual presentation were included (n=1930).
Table 3a. Motor severity in recent onset PD, in relation to vascular risk factors, restricted to 1620 cases without a history of stroke, TIA or cardiac disease. Results based on sensitivity analysis where individuals with unusual presentation were included.
Table 3b. Cognitive status in recent onset PD, in relation to vascular risk factors, restricted to 1620 cases without a history of stroke, TIA or cardiac disease. Results based on sensitivity analysis where individuals with unusual presentation were included.
Table 4. Cognitive and motor severity in 939 cases with structural brain imaging. Results based on sensitivity analysis where individuals with unusual presentation were included.

## References

[1] Hu MT , Szewczyk‐Krolikowski K , Tomlinson P , et al. Predictors of cognitive impairment in an early stage Parkinson's disease cohort. Mov Disord 2014;29:351–359. 24395708 10.1002/mds.25748 PMC4235340

[2] Pfeiffer HC , Lokkegaard A , Zoetmulder M , Friberg L , Werdelin L . Cognitive impairment in early‐stage non‐demented Parkinson's disease patients. Acta Neurol Scand 2014;129:307–318. 24117192 10.1111/ane.12189

[3] Wardlaw JM , Valdes Hernandez MC , Munoz‐Maniega S . What are white matter hyperintensities made of? Relevance to vascular cognitive impairment. Am Heart J 2015; 4:001140. 10.1161/JAHA.114.001140 PMC4599520 26104658

[4] Hajjar I , Quach L , Yang F , et al. Hypertension, white matter hyperintensities, and concurrent impairments in mobility, cognition, and mood: the Cardiovascular Health Study. Circulation 2011;123:858–865. 21321150 10.1161/CIRCULATIONAHA.110.978114 PMC3081662

[5] Verdelho A , Madureira S , Ferro JM , et al. Differential impact of cerebral white matter changes, diabetes, hypertension and stroke on cognitive performance among non‐disabled elderly. The LADIS study. J Neurol Neurosurg Psychiatry 2007;78:1325–1330. 17470472 10.1136/jnnp.2006.110361 PMC2095587

[6] Dearborn JL , Knopman D , Sharrett AR , et al. The metabolic syndrome and cognitive decline in the Atherosclerosis Risk in Communities study (ARIC). Dement Geriatr Cogn Disord 2014;38:337–46. 25171458 10.1159/000362265 PMC4201882

[7] Stebbins GT , Goetz CG , Burn DJ , Jankovic J , Khoo TK , Tilley BC . How to identify tremor dominant and postural instability/gait difficulty groups with the movement disorder society unified Parkinson's disease rating scale: comparison with the unified Parkinson's disease rating scale. Mov Disord 2013;28:668–670. 23408503 10.1002/mds.25383

[8] Alves G , Larsen JP , Emre M , Wentzel‐Larsen T , Aarsland D . Changes in motor subtype and risk for incident dementia in Parkinson's disease. Mov Disord 2006;21:1123–1130. 16637023 10.1002/mds.20897

[9] Rosano C , Brach J , Studenski S , Longstreth WT Jr , Newman AB . Gait variability is associated with subclinical brain vascular abnormalities in high‐functioning older adults. Neuroepidemiology 2007;29:193–200. 18043004 10.1159/000111582 PMC2824582

[10] Hashimoto M , Takashima Y , Uchino A , Yuzuriha T , Yao H . Dual task walking reveals cognitive dysfunction in community‐dwelling elderly subjects: the Sefuri brain MRI study. J Stroke Cerebrovasc Dis 2014;23:1770–1775. 24957316 10.1016/j.jstrokecerebrovasdis.2014.05.008

[11] Kotagal V , Albin RL , Muller ML , Koeppe RA , Frey KA , Bohnen NI . Diabetes is associated with postural instability and gait difficulty in Parkinson disease. Parkinsonism Relat Disord 2013;19:522–526. 23462483 10.1016/j.parkreldis.2013.01.016 PMC3607954

[12] Bohnen NI , Muller ML , Zarzhevsky N , et al. Leucoaraiosis, nigrostriatal denervation and motor symptoms in Parkinson's disease. Brain 2011;134:2358–2365. 21653540 10.1093/brain/awr139 PMC3155702

[13] Baumgart M , Snyder HM , Carrillo MC , Fazio S , Kim H , Johns H . Summary of the evidence on modifiable risk factors for cognitive decline and dementia: a population‐based perspective. Alzheimers Dement 2015;11:718–726. 26045020 10.1016/j.jalz.2015.05.016

[14] Evans JR , Mason SL , Williams‐Gray CH , et al. The natural history of treated Parkinson's disease in an incident, community based cohort. J Neurol Neurosurg Psychiatry 2011;82:1112–1118. 21593513 10.1136/jnnp.2011.240366

[15] Bloem BR , Grimbergen YA , Cramer M , Willemsen M , Zwinderman AH . Prospective assessment of falls in Parkinson's disease. J Neurol 2001;248:950–8. 11757958 10.1007/s004150170047

[16] Santiago JA , Potashkin JA . System‐based approaches to decode the molecular links in Parkinson's disease and diabetes. Neurobiol Dis 2014;72(Pt A):84–91. 24718034 10.1016/j.nbd.2014.03.019

[17] Hughes AJ , Daniel SE , Lees AJ . Improved accuracy of clinical diagnosis of Lewy body Parkinson's disease. Neurology 2001;57:1497–1499. 11673599 10.1212/wnl.57.8.1497

[18] Tomlinson CL , Stowe R , Patel S , Rick C , Gray R , Clarke CE . Systematic review of levodopa dose equivalency reporting in Parkinson's disease. Mov Disord 2010;25:2649–2653. 21069833 10.1002/mds.23429

[19] Emre M , Aarsland D , Brown R , et al. Clinical diagnostic criteria for dementia associated with Parkinson's disease. Mov Disord 2007;22:1689–1707; quiz 837. 17542011 10.1002/mds.21507

[20] The QRISK®2–2015 risk calculator. http://www.qrisk.org. Accessed April 4, 2016.

[21] Rubin DB . Inference and missing data. Biometrika 1976;63:581–592.

[22] Buchman AS , Leurgans SE , Nag S , Bennett DA , Schneider JA . Cerebrovascular disease pathology and parkinsonian signs in old age. Stroke 2011;42:3183–3189. 21885844 10.1161/STROKEAHA.111.623462 PMC3202031

[23] Chaudhari TS , Verma R , Garg RK , Singh MK , Malhotra HS , Sharma PK . Clinico‐radiological predictors of vascular cognitive impairment (VCI) in patients with stroke: a prospective observational study. J Neurol Sci 2014;340:150–158. 24680559 10.1016/j.jns.2014.03.018

[24] Verghese J , Ayers E , Barzilai N , et al. Motoric cognitive risk syndrome: multicenter incidence study. Neurology 2014;83:2278–2284. 25361778 10.1212/WNL.0000000000001084 PMC4277675

[25] Mahlknecht P , Kiechl S , Willeit J , Poewe W , Seppi K . Motoric cognitive risk syndrome: multicenter incidence study. Neurology 2015;85:388–389. 10.1212/01.wnl.0000470376.04336.ea PMC10687609 26215881

[26] Lloyd‐Jones D , Adams R , Carnethon M , et al. Heart disease and stroke statistics—2009 update: a report from the American Heart Association Statistics Committee and Stroke Statistics Subcommittee. Circulation 2009;119:480–486. 19171871 10.1161/CIRCULATIONAHA.108.191259

[27] Guan J , Pavlovic D , Dalkie N , et al. Vascular degeneration in Parkinson's disease. Brain Pathol 2013;23:154–164. 22897695 10.1111/j.1750-3639.2012.00628.x PMC8029297

[28] Spauwen PJ , van Boxtel MP , Verhey FR , et al. Both low and high 24‐hour diastolic blood pressure are associated with worse cognitive performance in type 2 diabetes: the Maastricht study. Diabetes Care 2015;38:1473–1480. 26016842 10.2337/dc14-2502

[29] Park K , Yasuda N , Toyonaga S , et al. Significant association between leukoaraiosis and metabolic syndrome in healthy subjects. Neurology 2007;69:974–978. 17538033 10.1212/01.wnl.0000266562.54684.bf

[30] Mak E , Dwyer MG , Ramasamy DP , et al. White matter hyperintensities and mild cognitive impairment in Parkinson's disease. J Neuroimaging 2015;25:754–760. 25753576 10.1111/jon.12230

[31] Beyer MK , Aarsland D , Greve OJ , Larsen JP . Visual rating of white matter hyperintensities in Parkinson's disease. Mov Disord 2006;21:223–229. 16161159 10.1002/mds.20704

[32] Vesely B , Rektor I . The contribution of white matter lesions (WML) to Parkinson's disease cognitive impairment symptoms: a critical review of the literature. Parkinsonism Relat Disord 2016;22(suppl 1):S166–S170. 26391185 10.1016/j.parkreldis.2015.09.019

[33] Slawek J , Wieczorek D , Derejko M , et al. The influence of vascular risk factors and white matter hyperintensities on the degree of cognitive impairment in Parkinson's disease. Neurol Neurochir Pol 2008;42:505–512. 19235103

[34] Meyer JS , Huang J , Chowdhury MH . MRI confirms mild cognitive impairments prodromal for Alzheimer's, vascular and Parkinson‐Lewy body dementias. J Neurol Sci 2007;257:97–104. 17316690 10.1016/j.jns.2007.01.016

[35] Slawek J , Roszmann A , Robowski P , et al. The impact of MRI white matter hyperintensities on dementia in Parkinson's disease in relation to the homocysteine level and other vascular risk factors. Neurodegener Dis 2013;12:1–12. 22831964 10.1159/000338610

[36] Lee SJ , Kim JS , Yoo JY , et al. Influence of white matter hyperintensities on the cognition of patients with Parkinson disease. Alzheimer Dis Assoc Disord 2010;24:227–233. 20473133 10.1097/WAD.0b013e3181d71a13

[37] Kandiah N , Mak E , Ng A , et al. Cerebral white matter hyperintensity in Parkinson's disease: a major risk factor for mild cognitive impairment. Parkinsonism Rel Disord 2013;19:680–3. 10.1016/j.parkreldis.2013.03.008 23623194

[38] Gonzalez‐Redondo R , Toledo J , Clavero P , et al. The impact of silent vascular brain burden in cognitive impairment in Parkinson's disease. Eur J Neurol 2012;19:1100–1107. 22360775 10.1111/j.1468-1331.2012.03682.x

[39] Bohnen NI , Kotagal V , Muller ML , et al. Diabetes mellitus is independently associated with more severe cognitive impairment in Parkinson disease. Parkinsonism Rel Disord 2014;20:1394–1398. 10.1016/j.parkreldis.2014.10.008 PMC4314515 25454317

[40] Chen YF , Tseng YL , Lan MY , et al. The relationship of leukoaraiosis and the clinical severity of vascular Parkinsonism. J Neurol Sci 2014;346:255–259. 25240444 10.1016/j.jns.2014.09.002

[41] Vesely B , Antonini A , Rektor I . The contribution of white matter lesions to Parkinson's disease motor and gait symptoms: a critical review of the literature. J Neural Transm (Vienna). 2015;123:241–250. 26483133 10.1007/s00702-015-1470-9

[42] Cereda E , Barichella M , Cassani E , Caccialanza R , Pezzoli G . Clinical features of Parkinson disease when onset of diabetes came first: a case‐control study. Neurology 2012;78:1507–1511. 22539572 10.1212/WNL.0b013e3182553cc9

[43] Rektor I , Goldemund D , Sheardova K , Rektorova I , Michalkova Z , Dufek M . Vascular pathology in patients with idiopathic Parkinson's disease. Parkinsonism Rel Disord 2009;15:24–29. 10.1016/j.parkreldis.2008.02.007 18403246

[44] Olanow CW , Wunderle KB , Kieburtz K . Milestones in movement disorders clinical trials: advances and landmark studies. Mov Disord 2011;26:1003–14. 21626545 10.1002/mds.23727

