# Supplementary material for: Vascular disease and vascular risk factors in relation to motor features and cognition in early Parkinson's disease
Source: Mov Disord. 2016 Oct 6;31(10):1518–26. doi: 10.1002/mds.26698 (PMC5082556; doi:10.1002/mds.26698)
Supplement: Supplementary file 1 — Table 2. Motor and cognitive profile classified by prior history of stroke or cardiac disease. Results based on sensitivity analysis where individuals with unusual presentation were included (n=1930). Table 3a. Motor severity in recent onset PD, in relation to vascular risk factors, restricted to 1620 cases without a history of stroke, TIA or cardiac disease. Results based on sensitivity analysis where individuals with unusual presentation were included. Table 3b. Cognitive status in recent onset PD, in relation to vascular risk factors, restricted to 1620 cases without a history of stroke, TIA or cardiac disease. Results based on sensitivity analysis where individuals with unusual presentation were included. Table 4. Cognitive and motor severity in 939 cases with structural brain imaging. Results based on sensitivity analysis where individuals with unusual presentation were included. [file MDS-31-1518-s001.docx]

Table 2. Motor and cognitive profile classified by prior history of stroke or cardiac disease. Results based on sensitivity analysis where individuals with unusual presentation were included (n=1930).

|  | Previous stroke or TIA | | Model Estimates^d^ (95% CI) |  | p value^d^ | | Cardiac disease | | | Model Estimates^d^ (95% CI) | | p value^d^ | |
| --- | --- | --- | --- | --- | --- | --- | --- | --- | --- | --- | --- | --- | --- |
| Characteristic | Yes | No |  |  | | Yes | | No |  | |  | |  |
|  | N=94  (4.9%) | N=1834  (95.1%) |  |  | | N=243  (12.7%) | | N=1676  (87.3%) |  | |  | |  |
| *Montreal cognitive assessment*^a^ | | |  |  | |  | |  |  | |  | |  |
| Normal | 52 (57.8%) | 1271 (74.4%) | 1.38^e^  (0.89, 2.16) | 0.15^f^ | | 146 (62.9%) | | 1171 (75.1%) | 1.16^e^  (0.86, 1.58) | | 0.33^f^ | |  |
| MCI | 35 (38.9%) | 384 (22.5%) |  |  | | 73 (31.5%) | | 345 (22.1%) |  | |  | |  |
| Dementia | 3 (3.3%) | 54 (3.2%) |  |  | | 13 (5.6%) | | 44 (2.8%) |  | |  | |  |
| UPDRS 3^b^ | 26.1 (11.7) | 22.7 (12.2) | 1.48^g^  (-1.21, 4.17) | 0.28^h^ | | 25.5 (13.1) | | 22.5 (12.1) | 1.41^g^  (-0.32, 3.15) | | 0.11^h^ | |  |
| *Motor phenotype*^c^ | |  |  |  | |  | |  |  | |  | |  |
| TD | 28 (34.1%) | 796 (46.7%) | 1^i^ (ref) |  | | 84 (38.4%) | | 737 (47.3%) | 1^i^ (ref) | |  | |  |
| PIGD | 43 (52.4%) | 688 (40.4%) | 1.58^i^  (0.96, 2.59) | 0.069 | | 106 (48.4%) | | 621 (39.8%) | 1.33^i^  (0.97, 1.83) | | 0.074 | |  |
| Indeterminate | 11 (13.4%) | 220 (12.9%) | 1.45^i^  (0.71, 2.99) | 0.31 | | 29 (13.2%) | | 201 (12.9%) | 1.32^i^  (0.83, 2.10) | | 0.24 | |  |

Abbreviations: TIA = transient ischemic attack, MCI = mild cognitive impairment, UPDRS 3 = Movement Disorder Society unified Parkinson’s disease rating scale part 3, TD = tremor dominant, PIGD = postural instability gait difficulty.

^a^ Ordinal logistic regression model (normal=0, MCI=1, Dementia=2)

^b^ Linear regression model

^c^ Multinomial logistic regression model with TD as baseline

^d^Adjusted for age, gender and disease duration

^e^ Odds ratio

^f^ Also adjusted for drug naïve

^g^ Beta coefficient (adjusted difference in means)

^h^ Also adjusted for LEDD

^i^ Multinomial odds ratio

Table 3a. Motor severity in recent onset PD, in relation to vascular risk factors, restricted to 1620 cases without a history of stroke, TIA or cardiac disease. Results based on sensitivity analysis where individuals with unusual presentation were included.

|  |  |  |  | UPDRS 3^a^ |  |
| --- | --- | --- | --- | --- | --- |
|  |  | Number (%) | Mean  (sd) | Beta^b^  (95% CI) | p value |
| *Vascular risk factors* | Cigarette Smoking | 65 (4.5%) | 22.9  (14.6) | 2.55  (-0.41, 5.52) | 0.091^c^ |
|  | Hypertension | 492 (30.5%) | 24.0  (12.1) | 0.53  (-0.94, 2.01) | 0.48^c^ |
|  | High cholesterol | 441 (27.3%) | 23.6  (12.3) | -0.08  (-1.59, 1.43) | 0.92^c^ |
|  | Diabetes Mellitus | 121 (7.5%) | 27.3  (14.7) | 3.09  (0.67, 5.52) | 0.013^c^ |
|  | BMI > 30 | 327 (20.7%) | 23.7  (11.7) | 1.73  (0.16, 3.29) | 0.031^c^ |
| *Number of vascular risk factors^d^* | None | 617 (44.3%) | 20.6  (10.7) | 0  (ref) | - |
|  | 1 | 439 (31.5%) | 21.9  (12.3) | 1.00  (-0.45, 2.46) | 0.18 |
|  | 2 | 236 (16.9%) | 22.2  (11.7) | 0.83  (-0.95, 2.61) | 0.36 |
|  | >2 | 102  (7.3%) | 25.6  (11.1) | 4.10  (1.64, 6.56) | 0.001 |
| *Vascular risk score* | QRISK2 >20 | 610  (38.0%) | 25.0  (12.7) | 3.16  (1.43, 4.89) | <0.001 |

Abbreviations PD = Parkinson’s disease, TIA = transient ischemic attack, UPDRS 3 = Movement Disorder Society unified PD rating scale part 3, MCI = mild cognitive impairment, OR = odds ratio, CI = confidence interval, BMI = body mass index.

^a^ Linear regression model, UPDRS score for all patients was mean 22.3 (SD 12.0)

^b^ Adjusted for age, gender, disease duration and LEDD; result is from comparison of cases with vascular risk to those without

^c^ Also mutually adjusted for all vascular risk factors

^d^ Restricted to complete casesTable 3b. Cognitive status in recent onset PD, in relation to vascular risk factors, restricted to 1620 cases without a history of stroke, TIA or cardiac disease. Results based on sensitivity analysis where individuals with unusual presentation were included.

|  |  |  | Cognitive status^a^ | | | |
| --- | --- | --- | --- | --- | --- | --- |
|  | Total | Normal  N (%)  1140 (75.8%) | MCI  N (%)  323 (21.5%) | Dementia  N (%)  42 (2.8%) | OR^b^  (95% CI) | p value |
| *Vascular risk factors* | Cigarette Smoking | 48 (77.4%) | 14 (22.6%) | 0 (0.0%) | 1.69  (0.89, 3.21) | 0.11^c^ |
|  | Hypertension | 326 (71.0%) | 116 (25.3%) | 17 (3.7%) | 1.04  (0.76, 1.42) | 0.81^c^ |
|  | High cholesterol | 281 (69.7%) | 110 (27.3%) | 12 (3.0%) | 1.21  (0.88, 1.66) | 0.24^c^ |
|  | Diabetes Mellitus | 63 (58.9%) | 32 (29.9%) | 12 (11.2%) | 1.90  (1.16, 3.11) | 0.011^c^ |
|  | BMI > 30 | 227 (75.2%) | 64 (21.2%) | 11 (3.6%) | 1.14  (0.80, 1.61) | 0.48^c^ |
| *Number of vascular risk factors ^d^* | None | 472 (81.7%) | 98 (17.0%) | 8 (1.4%) | 1  (ref) | - |
|  | 1 | 320 (78.8%) | 78 (19.2%) | 8 (2.0%) | 1.08  (0.78, 1.51) | 0.63 |
|  | 2 | 168 (75.0%) | 47 (21.0%) | 9 (4.0%) | 1.25  (0.85, 1.83) | 0.25 |
|  | >2 | 58 (64.4%) | 30 (33.3%) | 2 (2.2%) | 2.07  (1.27, 3.38) | 0.004 |
| *Vascular risk score* | QRISK2 >20 | 359 (63.5%) | 179 (31.7%) | 27 (4.8%) | 1.48  (1.04, 2.13) | 0.031 |

Abbreviations PD = Parkinson’s disease, TIA = transient ischemic attack, UPDRS 3 = Movement Disorder Society unified PD rating scale part 3, MCI = mild cognitive impairment, OR = odds ratio, CI = confidence interval, BMI = body mass index.

^a^ Ordinal logistic regression model

^b^ Adjusted for age, gender, disease duration and drug naïve

^c^ Also mutually adjusted for all vascular risk factors

^d^ Restricted to complete cases

Table 4. Cognitive and motor severity in 939 cases with structural brain imaging. Results based on sensitivity analysis where individuals with unusual presentation were included.

| Characteristic | Leukoaraiosis only | Brain CT or MR result Lacunar or territory infarct | No vascular disease | Model estimate^d^ Leukoaraiosis only vs no vascular disease (95% CI) | Model estimate^d^ Lacunar or territory infarct vs no vascular disease (95% CI) | p value^d,e^ |
| --- | --- | --- | --- | --- | --- | --- |
| Total | 136 (14.5%) | 108 (11.5%) | 695 (74.0%) |  |  |  |
| *Montreal cognitive assessment^a^* | | |  |  |  |  |
| Normal | 71 (56.8%) | 76 (76.0%) | 493 (75.8%) | 1.59^f^ (1.05, 2.40) | 0.71^f^ (0.42, 1.18) | 0.018^g^ |
| MCI | 42 (33.6%) | 23 (23.0%) | 133 (20.5%) |  |  |  |
| Dementia | 12 (9.6%) | 1 (1.0%) | 24 (3.7%) |  |  |  |
| UPDRS 3^b^ | 24.4 (11.5) | 24.1 (12.3) | 21.8 (11.7) | 1.29^h^ (-1.00,3.58) | 1.65^h^ (-0.91,4.21) | 0.30^i^ |
| *Motor phenotype^c^* | |  |  |  |  |  |
| TD | 38 (29.9%) | 45 (47.4%) | 270 (41.7%) | 1^j^ (ref) | 1^j^ (ref) |  |
| PIGD | 75 (59.1%) | 40 (42.1%) | 290 (44.8%) | 1.55^j^ (1.00,2.41) | 0.74^j^ (0.47, 1.18) | 0.039 |
| Indeterminate | 14 (11.0%) | 10 (10.5%) | 88 (13.6%) | 1.03^j^ (0.53, 2.03) | 0.64^j^ (0.31, 1.34) | 0.48 |

Abbreviations: CT = computed tomography, MR = magnetic resonance, MCI = mild cognitive impairment, UPDRS 3 = Movement Disorder Society unified PD rating scale, TD = tremor dominant, PIGD = postural instability gait difficulty

^a^ Ordinal logistic regression model (normal=0, MCI=1, Dementia=2)

^b^ Linear regression model

^c^ Multinomial logistic regression model with TD as baseline

^d^ Adjusted for age, gender and disease duration

^e^ Heterogeneity test p-value across the three groups

^f^ Odds ratio

^g^ Also adjusted for drug naïve

^h^ Beta coefficient (adjusted difference in means)

^i^ Also adjusted for LEDD

^j^ Multinomial odds ratio
